# A case series of children and young people admitted to a tertiary care hospital in Germany with COVID-19

**DOI:** 10.1186/s12879-021-05791-8

**Published:** 2021-02-01

**Authors:** Jonathan Remppis, Tina Ganzenmueller, Malte Kohns Vasconcelos, Oliver Heinzel, Rupert Handgretinger, Hanna Renk

**Affiliations:** 1 Department of Haematology and Oncology, University Children’s Hospital Tuebingen, Hoppe-Seyler-Straße 1, 72076 Tuebingen, Germany; 2 Institute for Medical Virology, University Hospital Tuebingen, Elfriede-Aulhorn-Str. 6, 72076 Tuebingen, Germany; 3 Institute for Medical Microbiology and Hospital Hygiene, Heinrich Heine University Düsseldorf, Düsseldorf, Germany

**Keywords:** COVID-19, SARS CoV-2 infection, Children, Comorbidity, Inpatients

## Abstract

**Background:**

While our knowledge about COVID-19 in adults has rapidly increased, data on the course of disease and outcome in children with different comorbidities is still limited.

**Methods:**

Prospective, observational study at a tertiary care children’s hospital in southern Germany. Clinical and virology data from all paediatric patients admitted with SARS-CoV-2 infection at our hospital were prospectively assessed.

**Results:**

Between March and November 2020, 14 patients were admitted with COVID-19. One patient was admitted a second time with COVID-19 6 months after initial disease. Among seven patients with severe underlying comorbidities, three developed multisystem inflammatory syndrome (MIS-C), two were admitted to the paediatric intensive care unit. One patient needed invasive ventilation. Another patient died shortly after discharge of COVID-19-related complications.

**Conclusions:**

While COVID-19 generally causes mild disease in children, severe respiratory illness and MIS-C occur, in some cases with fatal outcome. Children with underlying diseases might be at special risk for severe disease.

## Background

Severe acute respiratory syndrome coronavirus 2 (SARS-CoV-2) has caused the pandemic of coronavirus disease 2019 (COVID-19) with more than 50 million cases and over 1.3 million deaths globally until 15 November 2020 [1]. This outbreak challenges healthcare systems worldwide. Children generally develop milder disease than adults; the majority of infections are asymptomatic or manifest as upper respiratory tract infection or mild pneumonia [2–4]. Nevertheless, severe and fatal cases have been reported in children [5]. Furthermore, multisystem inflammatory syndrome in children (MIS-C) is increasingly observed among children with COVID-19 [6, 7]. Several risk factors for severe illness and mortality due to COVID-19 have been identified among adult patients, such as hypertension, diabetes and coronary heart disease [8]. However, our knowledge about the course of disease and outcome of COVID-19 in children with different comorbidities is still limited [9]. Pre-existing medical conditions, obesity and young age (< 1 year) appear to be risk factors for hospitalisation and paediatric intensive care unit (PICU) admission in children [5, 10–12].

However, immunosuppressive treatment has not been associated with severe course of disease [7, 11].

As a large number of patients with potential risk factors are treated at the University Children’s Hospital Tuebingen, we chose this site for further research on COVID-19 among children. The aim of this study was to assess clinical characteristics of children hospitalised for COVID-19 treatment at our hospital.

## Methods

### Hospital setting and participants

The University Children’s Hospital Tuebingen is a large tertiary care facility in southern Germany comprising departments for paediatric oncology and haematology, cardiology and pulmonology, surgery, neuropaediatrics, and neonatology. At the same time, it serves as regional primary and secondary care referral centre and provides treatment for a substantial number of high-risk patients, such as patients undergoing haematopoietic stem cell transplantation (HSCT), solid organ transplant (SOT) and thoracic, cardiac or vascular surgery.

### Screening and testing strategy

An overview of the SARS-CoV-2 testing and infection control strategies in our hospital during the first wave of the pandemic has been published [13].

### Data collection

Between March and November 2020 data from children admitted to our hospital with COVID-19 were collected prospectively. We used the International Severe Acute Respiratory and Emerging Infection Consortium (ISARIC) Clinical Characterisation Protocol for standardised clinical data collection, which was designed for any severe or potentially severe acute infection of public health interest [14, 15]. After obtaining written informed consent, data on demography, clinical presentation and course, treatment and outcome as well as virology results was pseudonymized and collected. Data was retrieved from hospital records and patient interviews. We used REDCap 10.0.3 (Vanderbilt University, Nashville, Tennessee, USA) and Microsoft Excel (Microsoft Excel 2010 v14.0, Microsoft Corporation, Redmond, Washington, USA) for data collection and analysis.

## Results

Between 29 February and 30 November 2020, a total of 3628 respiratory specimens were collected from patients aged 0–18 years and tested for SARS-CoV-2 ribonucleic acid (RNA) at the University Children’s Hospital Tuebingen. These correspond to 3157 outpatient visits and hospital admissions of a total of 2295 patients.

SARS-CoV-2 infection was present in 14 patients admitted to our children’s hospital with COVID-19 between 26 March and 30 November. Of note, one of these patients was tested positive for a second time. He was re-admitted with COVID-19 infection 6 months after initial hospital treatment. Another patient was readmitted for other reasons, without symptoms of COVID-19, 4 weeks after initial treatment and still found weakly positive for SARS-CoV-2-RNA in throat swabs.

All patients were admitted to the paediatric COVID 19/respiratory infections ward. In 13 cases SARS-CoV-2 infection was detected on admission, in 2 cases prior to admission. Data on demography, clinical course, treatment and outcome are presented in Table 1.

**Table 1:** Paediatric patients admitted with COVID-19

N°	Age Range (years)	Sex	Past medical history	Clinical presentation				Laboratory findings			Infiltrates in X-ray or CT	Treatment				Co-Infections	Complications ^**b)**^	MIS-C	Outcome
				Fever	Respiratory Symptoms	Gastrointestinal Symptoms	Other Symptoms	Max. CRP (mg/dl)	Max. D-Dimer (μg/ml)	Max. Troponin (ng/ml)		PICU treatment	Oxygen support	Invasive ventilation	Pharmaco-logical treatment ^**b)**^				
1	< 1	m	Healthy	X		X		Neg	NA	NA	NA					None	None		Complete recovery
2	≥ 16	w	Kidney transplantation 2008, immunosuppressive treatment	X	X	X		38	50	507	Yes	X	X	X	Antibiotic, antifungal, heparin	None	Pneumonia, pleural effusion, acute renal failure, hypertension	X	Complete recovery
3	≥ 16	m	Hypoplastic left heart syndrome		X		Hypoxaemia	15	6	Neg	No	X	X		None	None	Cerebral vasculitis, myocarditis	X	Died 11 days after discharge
4	6–12	m	Oesophageal atresia, recurrent otitis media, multiple interventions	X		X	Orbital swelling	16	5	Neg	NA				Antibiotic	NA	None		Mild residual symptoms
5 = 15	6–12	m	Rhabdomyosarcoma, cytotoxic chemotherapy		X			5	Neg	Neg	NA				Antibiotic	None	None		Complete recovery, Reinfection
6	6–12	w	Healthy	X	X	X		16	5	14	Yes		X		Antibiotic, heparin, defibrotide	None	Terminal ileitis, pneumonia, polyserositis	X	Complete recovery
7	≥ 16	w	Severe immunodeficiency syndrome with T-cell-disorder, HSCT 2019	X	X	X		14	1	Neg	Yes		X		Antibiotic, antifungal, ribavirin, valganciclovir	Throat swab: HSV-1, CMV, EBV, HHV-6, *Enterobacter cloacae*	Pneumonia, pericardial effusion	X	Complete recovery
8	< 1	w	Healthy				Livid discoloration of the legs	2	2	NA	NA				None	NA	None		Mild residual symptoms
9	6–12	w	Asthma, obesity	X	X			Neg	Neg	Neg	NA				Inhaled beta-2-mimetics	NA	None		Complete recovery
10	13–15	m	Sickle cell anaemia, Asthma		X		Haematuria	6	7	Neg	No		X^a)^		Antibiotic, heparin	NA	None		Mild residual symptoms
11	13–15	m	Healthy	X	X	X	Haemat-emesis	13	1	Neg	Yes				Antibiotic, heparin	Sputum: *Streptococcus pneumoniae*	Bacterial pneumonia		Mild residual symptoms
12	< 1	m	Healthy	X	X			Neg	NA	NA	NA				Antibiotic	None	None		complete recovery
13	1–2	w	Chronic Bronchitis	X	X			2,5	Neg	Neg	NA				None	NA	None		complete recovery
14	≥ 16	w	Liver transplantation 2019, immunosuppressive treatment		X	X		6	NA	NA	NA				None	NA	None		complete recovery
15 = 5	6–12	m	Rhabdomyosarcoma, cytotoxic chemotherapy		X			Neg	Neg	Neg	NA				None	NA	None		Mild residual symptoms

*Abbreviations*: *CMV* cytomegalovirus, *CRP* C-reactive protein, *CT* computed tomography, *EBV* Epstein-Barr virus, *F* female, *HHV-6* human herpesvirus 6, *HSCT* haematopoietic stem cell transplantation, *HSV* herpes simplex virus, *M* male, *MIS-C* multisystem inflammatory syndrome in children, *neg* negative, *NA* not assessed, *PICU* paediatric intensive care unit

^a^Therapeutic oxygen without respiratory impairment; ^b^COVID-19 related

### Course of disease

Median time from symptom onset to admission was 2 days (Interquartile range (IQR) 1; 4). Fever was present in 9/15 cases, respiratory symptoms (cough, dyspnoea, sore throat, rhinitis) were present in 12/15 cases. Gastrointestinal symptoms (diarrhoea, vomiting, abdominal pain) were present in 7/15 cases, in 2 cases without concomitant respiratory symptoms. One patient presented with orbital swelling and another one with livid discoloration of the legs as the initial symptom. Pulmonary infiltrates in chest x-ray or computed tomography were present in 4/15 cases. C-reactive protein was elevated during the course of disease in 11/15 cases with a median peak of 13 mg/dl (IQR 5.5; 15.5). D-Dimer elevation was detected in 8/15 cases. Among these, two underwent thromboelastometry; one had a pathologic result with increased activation of blood clotting. Troponin was elevated in two cases. Complications related to COVID-19 were observed in 5/15 cases: four had pneumonia or pleural effusions, four had cardiac involvement, and one had severe bowel inflammation. According to the preliminary definition by the World Health Organization [16], 4/15 cases in our cohort were diagnosed with MIS-C. Oxygen therapy was required in 4/15 cases and two patients needed PICU admission. Median hospital length of stay (LOS) was 5 days (IQR 2; 11.5).

### Course of disease in patients with underlying risk factors

In 13 out of our 15 cases, at least one of the above mentioned risk factors was present: three patients were infants younger than 1 year. Four patients were underweight, and one was obese. Nine patients had underlying comorbidities, of which seven were severe. Severe comorbidities comprised an impaired immune system in five patients due to immunosuppressive treatment after SOT (*n* = 2), cytotoxic chemotherapy (*n* = 1), after HSCT (*n* = 1) and due to sickle cell anaemia (*n* = 1). Severe comorbidities other than immunosuppression were present in two patients: One boy had a hypoplastic left heart syndrome and had undergone Norwood –and Glenn-procedure but was still in need of home oxygen treatment. The other boy had undergone multiple surgeries due to oesophageal atresia and adenoid vegetations, but had completed his surgical treatment at the time of COVID-19 infection.

Among these seven patients with severe underlying health conditions, three had MIS-C and accounted for a proportion of 75% of all our MIS-C patients (*n* = 4) so far. Two of these three patients were immunosuppressed (post HSCT and SOT) and the other patient was the boy with hypoplastic left heart syndrome. The girl with HSCT due to severe immunodeficiency syndrome with T-cell disorder required oxygen supplementation via nasal cannula only, whereas the girl who had received kidney transplantation 12 years previously was mechanically ventilated over 7 days. The boy with hypoplastic left heart syndrome received oxygen therapy with an increased flow volume of maximum six litres/minute via oral-nasal mask.

The patient with sickle cell anaemia developed a sickle cell crisis with gross haematuria concomitant to COVID-19 and received therapeutic oxygen without respiratory impairment.

The patient who had recently received cytotoxic chemotherapy due to a rhabdomyosarcoma had a sore throat as the only symptom of COVID-19, but was treated with intravenous antibiotics due to chemotherapy-related neutropenia. In his case, after detection of SARS-CoV-2 infection by a single positive polymerase chain reaction (PCR) test, multiple follow up PCR tests (the first retrieved after 8 days) were negative. Six months later the patient was readmitted with dry cough and headache, this time two PCR tests taken in a 9 day interval were positive for SARS-COV-2-RNA.

Median hospital LOS among the patients with severe underlying health conditions was 9 days (IQR 5; 12.5). The two patients admitted to the PICU stayed there 9 and 10 days, respectively.

### Treatment and outcome

Antibiotic treatment was administered in 8/15 cases, low-dosed heparin therapy in 4/15 cases. Experimental treatments were used in two patients: the patient with coagulation disorder received treatment with defibrotide and low-dosed heparin to prevent blood clotting. Under treatment, thromboelastometry returned to normal after 8 days. A detailed report about her course of disease has been published [17]. Ribavirin was administered to the girl who had undergone HSCT a year before.

In all 15 cases, patients were discharged alive and with no or only mild residual symptoms. However, the 18-year-old boy died at home, 11 days after discharge. Autopsy of the brain showed diffuse cerebral vasculitis of leptomeningeal und parenchymal blood vessels and myocarditis, both presumably associated with COVID-19 [18, 19].

## Discussion

Given the reported complications of COVID-19 such as acute respiratory distress syndrome, cardiac involvement, thromboembolic complications, exuberant inflammatory response, renal failure and secondary infections [8], some of the patient groups seeking specialised care at a tertiary care children’s hospital might be at increased risk of severe disease or death due to COVID-19. Among the 14 patients admitted to our hospital with COVID-19, 9 had underlying health conditions, which is more than reported in comparison to other tertiary or quaternary paediatric hospitals [11]. This might point at an elevated risk of severe disease among patients with comorbidities. Other explanations would be the generally increased proportion of chronically ill patients affiliated to our hospital or a lower threshold for hospital admission in these patients.

The increased rate of respiratory insufficiency and PICU admission among the chronically ill patients in our cohort is in line with literature reports of more severe disease in children with pre-existing medical conditions [5, 11]. The rate of 27% MIS-C in our cohort was comparable to a recent report from New York by Fernandes et al. [7]. Although our overall number of MIS-C patients is limited, we observed a high rate of comorbidities in these patients. This has not been reported so far [7, 11]. Furthermore, more than a third of our COVID-19 cohort suffered from an immunosuppressive condition. Immunosuppression has not been found a risk factor for severe COVID-19 in children in other reports [7, 11]. However, it has recently been postulated that prior Human Coronavirus (HCoV) infection possibly modifies COVID-19 severity and could be responsible for the relative protection from COVID-19 in children [20]. Therefore, it might be well possible that children with underlying medical conditions are generally less exposed to HCoV infections due to a more cautious health and hygiene behaviour (e.g. wearing masks when immunosuppressed). Reduced prior exposure to HCoV could then, in return, contribute to a more severe course of COVID-19 in immunosuppressed children.

In the two cases with atypical clinical presentation (patients number four and eight) it remains unclear, whether infection with SARS-CoV-2 has been causative or an incidental finding, as asymptomatic infection with SARS-CoV-2 is a common finding among young children [21]. While circulatory disorders due to increased blood clotting have been repeatedly reported in COVID-19 patients [7, 22], only a single case report described orbital cellulitis in the context of COVID-19 [23].

The patient with persistence of SARS-CoV-2-RNA in the throat 4 weeks after infection was under immunosuppressive treatment. In her case we presume persistence of RNA excretion rather than re-infection because of decreasing cycle of quantification (C_q_) values.

In the other patient readmitted with COVID-19 six months after initial disease, it remains unclear whether re-infection or persistence of viral RNA excretion is causative. In retrospective, we can also not exclude a false positive initial finding; however, viral respiratory testing was otherwise negative. Re-infection with SARS-CoV-2 has been repeatedly described in adults [24]. Although no reports from re-infected children have been published so far, it is not unexpected that immunity against SARS-CoV-2 may also wane in children. Persistence of viral RNA excretion up to 140 days, on the other hand, has been described among immunocompromised patients, particularly after B-cell-depletion [25, 26]. The patient in our cohort had undergone cytotoxic chemotherapy and bone directed radiotherapy, both leading to impaired viral immunity [27]. By the time of initial SARS-CoV-2 infection, B-cells were nearly completely depleted with a count of 2/μl.

The median duration of 2 days between symptom onset and hospital admission in our cohort was in line with reports from children but shorter than in adults [11, 28]. Similarly, hospital length of stay is comparable to other reports from children [7], but shorter than in adults [7, 14].

### Limitations

Despite a relatively high number of paediatric patients tested (*n* = 2295), the small number of children admitted with COVID-19 at our hospital limits strong conclusions on risk factors, clinical course and outcome. Due to the high proportion of special patient groups treated at our hospital, the observed cohort might not be representative for the general population and conclusions for other settings have to be drawn with caution.

## Conclusions

While COVID-19 generally causes mild disease in children, cases of severe respiratory disease and the development of MIS-C occur. Children with underlying health conditions seem to be more prone to these severe courses of disease. Although it remains unclear whether immunosuppressed patients have an increased risk of severe disease, they seem to be at risk for extended viral shedding and/or re-infection. Risk factors for severe courses of COVID-19 in children still remain to be elucidated. Further research is needed to improve disease prevention and management of COVID-19 in children with underlying medical conditions.

## Data Availability

No reprints are available. Data assessed from children admitted with COVID-19 was shared with ISARIC. A detailed report about patient N° 6 has been published in the Journal of the Pediatric Infectious Diseases Society [17]. A case report about patient N° 3 is currently in progress, but not yet submitted to a journal (Kumpf M. and Bösmüller H., unpublished data). The datasets used and/or analysed during the current study are available from the corresponding author on reasonable request.

## Abbreviations

SARS-CoV-2: Severe acute respiratory syndrome coronavirus 2
COVID-19: Coronavirus disease 2019
MIS-C: Multisystem inflammatory syndrome in children
PICU: Paediatric intensive care unit
HSCT: Haematopoietic stem cell transplantation
SOT: Solid organ transplant
ISARIC: Severe Acute Respiratory and Emerging Infection Consortium
RNA: Ribonucleic acid
IQR: Interquartile range
LOS: Length of stay
PCR: Polymerase chain reaction
HCoV: Human coronavirus
